# How Abnormal Is the Behaviour of Captive, Zoo-Living Chimpanzees?

**DOI:** 10.1371/journal.pone.0020101

**Published:** 2011-06-16

**Authors:** Lucy P. Birkett, Nicholas E. Newton-Fisher

**Affiliations:** School of Anthropology and Conservation, University of Kent, Canterbury, United Kingdom; Texas A&M University, United States of America

## Abstract

**Background:**

Many captive chimpanzees (*Pan troglodytes*) show a variety of serious behavioural abnormalities, some of which have been considered as possible signs of compromised mental health. The provision of environmental enrichments aimed at reducing the performance of abnormal behaviours is increasing the norm, with the housing of individuals in (semi-)natural social groups thought to be the most successful of these. Only a few quantitative studies of abnormal behaviour have been conducted, however, particularly for the captive population held in zoological collections. Consequently, a clear picture of the level of abnormal behaviour in zoo-living chimpanzees is lacking.

**Methods:**

We present preliminary findings from a detailed observational study of the behaviour of 40 socially-housed zoo-living chimpanzees from six collections in the United States of America and the United Kingdom. We determined the prevalence, diversity, frequency, and duration of abnormal behaviour from 1200 hours of continuous behavioural data collected by focal animal sampling.

**Results, Conclusion and Significance:**

Our overall finding was that abnormal behaviour was present in all sampled individuals across six independent groups of zoo-living chimpanzees, despite the differences between these groups in size, composition, housing, etc. We found substantial variation between individuals in the frequency and duration of abnormal behaviour, but all individuals engaged in at least some abnormal behaviour and variation across individuals could not be explained by sex, age, rearing history or background (defined as prior housing conditions). Our data support a conclusion that, while most behaviour of zoo-living chimpanzees is ‘normal’ in that it is typical of their wild counterparts, abnormal behaviour is endemic in this population despite enrichment efforts. We suggest there is an urgent need to understand how the chimpanzee mind copes with captivity, an issue with both scientific and welfare implications.

## Introduction

Captive conditions are known to evoke abnormal behaviour patterns in a variety of species, including non-human primates [1], [2], [3], [4]. Many chimpanzees (*Pan troglodytes*) kept in laboratory housing settings show a variety of serious behavioural abnormalities, such as repetitive rocking, drinking of urine, or self-mutilation [4], [5], [6], [7], [8], [9], [10], [11], [12]. Previous work indicates that various abnormal behaviour patterns also occur among chimpanzees held in zoological collections [13], [14], [15], [16], but detailed, quantitative studies on the zoo population are few [17].

Abnormal behaviours may indicate psychological suffering [18], but this is seldom considered directly (cf. Bradshaw and colleagues [11], [12]). Principles of evolutionary psychiatry [19] suggest that some abnormal behaviours may be symptomatic of underlying mental illness, a neglected area of research in great apes [17]. These possibilities raise serious ethical questions, particularly given the emotional and cognitive abilities of chimpanzees [20], [21], [22], [23], [24], although a consistent framework for the study of abnormal behaviour in apes has not yet been achieved [5], [17].

Social deprivation, and particularly maternal separation, have been suggested as causal factors in the development of abnormal behaviour in captive chimpanzees [15], [25], [26]: such events may be psychologically traumatic, or may deprive individuals of opportunities to learn appropriate behaviour. Persistent effects as a consequence of rearing have been found in some chimpanzees [16], but not all [27]. The impact of rearing history, while marked in younger chimpanzees, appears to wane as individuals age, at least in those re-socialised and housed with conspecifics [15], [27]. Such findings might suggest that social group housing, with the consequent opportunities for development of appropriate social relationships, will ameliorate the negative effects of early experiences [16], [28]. Social housing has been suggested as the most effective means of combating the occurrence and development of abnormal behaviours in primates [28], despite a lack of consensus over the causal roots of such behaviour [29], [30].

In this paper we present preliminary findings from a detailed, quantitative investigation of abnormal behaviour in captive (zoo-living) chimpanzees based on extensive and direct observation, comparable with that used for behavioural study of wild chimpanzees (for example: [31], [32], [33], [34]). We focus on adult and adolescent individuals housed in social groups, the context in which individuals should be least likely to display abnormal behaviour: our specific aim is to determine the level of abnormal behaviour in zoo-living, socially enriched, captive chimpanzees. We report detailed descriptive data on the occurrence of abnormal behaviour, and test whether performance of abnormal behaviour is related to social group size, rearing history or background (housing prior to entering the study group). As chimpanzee behaviour varies with age and sex, and some sex differences in abnormal behaviour have been reported [29], [35], we also test for the influence of these variables on the occurrence of abnormal behaviour.

## Materials and Methods

Abnormal behaviours among captive animals are those that are atypical of wild-living individuals, whether absent or occurring only rarely. We used published sources on behaviour in chimpanzees [6], [9], [11], [15], [29], [36], [37], [38] to compile a list of abnormal behaviours seen in the captive population. As a consequence of our observations (see below), ten further behaviours were added to this list: *bounce*, *groom stereotypically*, *groom stereotypically with object*, *move hand repetitively*, *rub hands repetitively*, *toss head, touch urine stream*, *poke anus*, and *walk on object*; *bite-hit-lick* was added for one individual who performed these behaviours as an integrated pattern. We used the most comprehensive ethogram for wild chimpanzees available [38] to determine which of the behaviours have been recorded for wild chimpanzees, and for these, to confirm that they occurred only rarely. Our final list consisted of 37 behaviours (Table 1).

**Table 1:** Abnormal behaviours used in this study.

Abnormal behavioural pattern and summary definition	Source of definition
*Bang self against surface* - hit own body-part against solid surface.	1
*Bite self* - bite own body-part.	1
*Bite-hit-lick self* - bite, hit, and lick own body-parts in constellation.	8
*Bounce* - bounce body or head, in non-social context.	8
*Clap* - slap palm of hand or sole of foot, making noise.	1
*Clasp self* - clutch own body.	1
*Configure lips* - move lips stereotypically, such as repetitively blowing air through lips or wiping lips over glass.	1
*Display to human* - stylized agonistic display and build-up directed at humans.	6
*Drink urine* - drink own urine.	1
*Eat faeces* - ingest own faeces, both matrix and undigested items (coprophagy).	1
*Eat faeces of other* - ingest another's faeces, both matrix and undigested items.	1
*Float limb* - entire limb (not just fingers or toes) appears to move independently, as if it does not belong to individual.	1, 5
*Fumble nipple* - manipulate own nipple(s) with thumb or fingers. May suck on nipple if breast or nipple is extended.	7, 8
*Groom stereotypically* - repetitively groom self on specific body part, seemingly without goal.	8
*Groom stereotypically with object* - groom self seemingly without intention, with stick or other tool. Drag object lightly over hairless body surface in non-focused way.	8
*Hit self* - hit own body-part with hand.	1
*Incest* - copulate with immediate relative.	7, 8
*Jerk* - spontaneously jerk body, apparently unprovoked by external stimulus.	5
*Manipulate faeces* - hold, carry, or spread own or other's faeces on surface.	1
*Move hand repetitively* - repetitively move hand in circular fashion in air or through substrate	8
*Pace* - locomote, usually quadrupedally, on substrate, covering and then re-covering route in stylized fashion, with no clear objective.	2
*Pat genitals* - repetitively touch own genitals, then often lick hand.	1
*Pinch self* - compress own skin between thumb and forefinger.	8
*Pluck hair* - pull out own hair.	1
*Pluck hair of other* - pull out another's hair.	1
*Poke anus* - insert finger into own anus.	8
*Poke eye* - poke one or more fingers into own eye.	1
*Regurgitate* - vomit voluntarily, then usually re-ingest vomitus.	1
*Rock* - sway repetitively and rhythmically, without piloerection. Usually side-to-side movement, but may be forward and backward or full circular motion of torso. Usually whole body, sometimes just the head.	1
*Rub hands* - run one hand over the other, then repeat but reverse the hand order, in stylized fashion.	8
*Spit* - expel saliva through pursed lips, often directed at human observer.	3, 4
*Stimulate self stylized, no context* - repetitively stroke or fondle own penis or clitoris in non-mating context.	1
*Toss head* - circular movement of head.	8
*Touch urine stream* - place hand or foot in own urine stream. May wipe hand on body after.	8
*Twirl* - rotate torso on axis for 360 degrees while upright and bipedal.	8
*Twitch body-part* - body-part, often fingers and toes, twitch repetitively, apparently involuntary.	5
*Walk on object* - locomote bipedally while carrying object (e.g. blanket), stepping on object edges that drag on floor. Individual swaggers to keep feet off ground and to maintain contact with object.	8

Sources: 1. Walsh et al. (1982); 2. Bloomsmith & Lambert (1995); 3. Nash et al. (1999); 4. Martin (2002); 5. Bradshaw et al. (2008); 6. Pederson et al. (2005); 7. Nishida et al. (2010); 8. This study.

The first author collected observational data on 40 chimpanzees living in six accredited zoological institutions (accrediting agencies were the American Zoological Association – AZA - and the British and Irish Associations of Zoos and Aquariums - BIAZA). The chimpanzees were all housed in social groups, with one group from each of the six participating zoological gardens contributing to our dataset. We chose the zoos from a master list that gave no information about the chimpanzees' or the institutions' characteristics, housing, or husbandry. Once selected, the participating zoos provided information on the background of their chimpanzees. Zoos often provide a valuable retirement sanctuary for primates and as a result, the backgrounds of the chimpanzees in this study were varied, including “wild caught,” entertainment industry, pet trade, laboratories, other zoological gardens, and sometimes a combination of these. As only partial background information was available in several cases, we classified background as either: *wild*, *laboratory*, *pet-trade/entertainment*, or *zoo*.

Across the groups there were 17 male and 23 female chimpanzees. Median group size was 6.5 (range = 3–11). Groups were either single-male (two groups) or multi-male (four groups, one of which was male only) and included a wide range of ages (Table 2). For analysis, we assigned individuals to age categories: 8–15 yrs, 16–23 yrs, 24–31 yrs, 32–39 yrs, and 40+ yrs. Five of the six groups showed a mix of rearing styles (*wild-born*, *mother-reared*, *hand-reared*: reared by human care-giver(s) and bottle fed); the sixth consisted of individuals who were all *hand-reared*. All groups had access to multiple areas (both indoor and outdoor). Only one of the groups had access restricted to a single areas at any one time, typically outside during the day, and inside at night. All enclosures met AZA/BIAZA standards.

**Table 2:** Study groups: size and composition.

Group	Group Size	Males	Females	Age range (years, category limits)
A	5	1	4	8–31
B	4	1	3	32–40+
C	8	4	4	8–39
D	9	2	7	8–39
E	3	3	0	24–39
F	11	6	5	8–40+

Groups were drawn from six accredited zoological institutions in the USA and UK; the behaviour of all group members was sampled (n = 40 individuals).

Data were collected from December 2008 to May 2010. Thirty hours of continuous focal sampling (a method of data collection in which all activities of a designated individual are recorded [39], [40]) were collected for each chimpanzee, yielding a total of 1200 observation hours (1800 min per individual). Data were collected using pen-and-paper from the public viewing areas at each zoo at distances ranging from 0.1 m (through glass) to about 10 m between observer and subject. Samples lasted 30 min and were collected in randomized rotation among members of each group, except for one zoo where reduced observability of subjects meant that samples lasted 20 min and could not be randomized at some times of the day. Once a focal sample began, recording was continuous unless a subject went out of sight; for every minute of such ‘bad observation’ one minute was added to the end of the sample.

From these data, we constructed four measures of abnormal behaviour: prevalence (proportion of individuals in a group who show a type of behaviour), diversity (number of types of behaviour), frequency (number of bouts of behaviour), and duration (amount of time spent performing a behaviour). Since our behavioural data were not normally distributed, we used nonparametric statistical tests [41]. All tests were two-tailed, and following convention, we set α at 0.05. Data were analyzed using SPSS Statistics 19 (SPSS Inc.).

## Results

**Figure 1:**
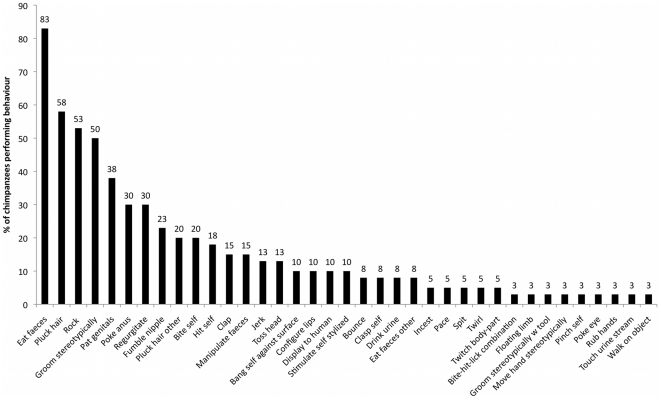
Percentage of chimpanzees from six independent zoological collections displaying each of the indicated abnormal behaviours.

All 40 chimpanzees showed some abnormal behaviour. Across groups, the most prevalent behaviour (0.83) was *eat faeces* (Table 3; Figure 1). Six behaviours were present in all six groups (*eat faeces*, *rock*, *groom stereotypically*, *pat genitals*, *regurgitate*, *fumble nipple*) and a further two (*pluck hair* and *hit self*) were present in five of the six groups. *Bite self* was shown by eight individuals across four of the groups. Across groups and behaviours, the median prevalence was 0.1 (range: 0.03–0.83). The number of different abnormal behaviours displayed in each group varied from 15 to 23 (mean = 18) and was not correlated with the number of individuals in the group (r = 0.46; n = 6, p = 0.35).

**Table 3:** Abnormal behaviours shown by zoo-living chimpanzees (n = 40).

		Duration		Frequency	
Behavioural Pattern	Prevalence	Median	Range	Median	Range
Eat faeces	0.83	13	1–53	5	1–16
Pluck hair	0.58	8	1–125	3	1–83
Rock	0.53	2	1–574	5.5	1–51
Groom stereotypically	0.50	20	1–172	9	1–152
Pat genitals	0.38	2	1–44	2	1–46
Poke anus	0.30	2	1–15	1.5	1–7
Regurgitate	0.30	5	1–33	2	1–35
Fumble nipple	0.23	1	1–173	2	1–56
Pluck hair other	0.20	6	1–106	3.5	1–24
Bite self	0.20	3	1–55	1	1–22
Hit self	0.18	1	1–42	1	1–10
Clap	0.15	1		4.5	1–10
Manipulate faeces	0.15	2	1–13	1.5	1–13
Jerk	0.13	1	1–3	3	1–200
Toss head	0.13	1		10	1–39
Bang self against surface	0.10	1	1–3	1	1–16
Configure lips	0.10	10	1–995	5	1–183
Display to human	0.10	5	2–13	2.5	1–7
Stimulate self stylized	0.10	9	3–15	4	2–38
Bounce	0.08	3	1–45	5	1–17
Clasp self	0.08	1		1	1–2
Drink urine	0.08	1	1–4	1	1–7
Eat faeces other	0.08	2	1–5	1	1–2
Incest	0.05	3	3–4	21	16–26
Pace	0.05	33	2–64	63	2–124
Spit	0.05	1		3	2–4
Twirl	0.05	1		15.5	1–30
Twitch body-part	0.05	47	45–49	33	25–41
Bite-hit-lick combination	0.03	139		29	
Floating limb	0.03	1		1	
Groom stereotypically w object	0.03	79		19	
Move hand stereotypically	0.03	141		35	
Pinch self	0.03	22		3	
Poke eye	0.03	66		230	
Rub hands	0.03	3		8	
Touch urine stream	0.03	4		25	
Walk on object	0.03	35		60	

*Prevalence* = number of individuals performing the behaviour/total number of individuals; *duration* = minutes/30 hrs observation; *frequency* = number of performances per 30 hrs observation. Abnormal behaviours were not mutually exclusive categories, and in some cases two or more occurred at the same time; *duration* is specific to each individual behaviour. Range only stated where min. and max. values differ.

Each of the 40 focal subjects displayed at least 2 abnormal behaviours (median: 5, range: 2–14). The number of different behaviours per individual (diversity) did not differ significantly across groups (Kruskal-Wallis H = 6.28, df = 5, p = 0.28). The lowest-scoring group had a median diversity of 4.5, and the highest-scoring group had a median diversity of 6.5. Similarly, the frequency of abnormal behaviour did not differ across groups (H = 5.75, df = 5, p = 0.33): group (C) with the lowest frequency had a median frequency of 0.6/hr, while group A had the highest median frequency of 3.2/hr (Table 4). The median total duration of abnormal behaviour likewise did not differ across groups (H = 1.28, df = 5, p = 0.94).

**Table 4:** Performance of 37 abnormal behaviour patterns by social group.

		Diversity		Frequency per 30 hr		Duration (%)	
Group	Total	Median	Range	Median	Range	Median	Range
A	20	6	5–11	97	20–482	3.5	0.9–62.4
B	15	6.5	3–8	34.5	13–320	4.0	1.9–7.7
C	16	4.5	3–6	17.5	5–190	2.9	1.2–35.5
D	17	5	2–9	34	6–109	4.2	1.1–25.3
E	18	6	5–14	30	24–105	4.3	2.8–5.5
F	23	5	3–12	48	5–260	7.6	0.1–11.9

Total = number of abnormal behavioural patterns observed in the group; diversity = number of abnormal behaviour patterns per individual; frequency = number of bouts per individual; duration = percentage of observation time spent performing abnormal behaviour per individual.

In contrast, both frequency and duration of abnormal behaviours varied greatly between individuals. Across individuals, the median frequency was 1.45/hr but this ranged from 0.13/hr to 13.5/hr. The median total duration of abnormal behaviour was 79 min, or 4.4% of 30 hr observation time, but there was great individual variation (0.1–62.4% of activity: Table 4)

We found no effect of age or sex on the occurrence of abnormal behaviour whether measured by diversity (Sex: Mann-Whitney U = 187, n = 17, 23, p = 0.811; Age: Kruskal-Wallis H = 4.40, df = 4, p = 0.35), frequency (Sex: U = 212, p = 0.652; Age: H = 7.11, df = 4, 0.13) or duration (Sex: U = 147, p = 0.185; Age: H = 5.75, df = 4, p = 0.22). We also found no effect of rearing history (*wild-born*: n = 9, *mother-reared*: n = 17, *hand-reared*: n = 9) on the occurrence of abnormal behaviour, for diversity (H = 1.95, df = 2, p = 0.38), frequency (H = 2.46, df = 2, p = 0.29) or duration (H = 0.05, df = 2, p = 0.97); the ‘unknown’ group (n = 5) was omitted from this analysis. We also found no effect of background (*wild*, *laboratory*, *pet-trade/entertainment*, *zoo*) on diversity (H = 2.92, df = 3, p = 0.40), frequency (H = 0.99, df = 3, p = 0.81), or duration (H = 0.74, df = 3, p = 0.86).

## Discussion

Our overall finding was that abnormal behaviour was present in all sampled individuals across six independent groups of zoo-living chimpanzees, despite the differences between these groups in size, composition, housing, etc. We found substantial variation between individuals in the frequency and duration of abnormal behaviour, but all individuals engaged in at least some abnormal behaviour, and variation across individuals could not be explained by sex, age, rearing history or background (defined as prior housing conditions).

Direct comparison of our results to earlier studies is complicated by differences in methods, particularly the measures used. Some studies coded only presence or absence for a behaviour, limiting any comparison to prevalence. On this measure, we found - across individuals and behaviours - a very similar level (0.10) to that (0.11; calculated from their Table 3) reported by Fritz et al. [10] (also Nash et al. [29]), although the most prevalent behaviour across these studies - *eat faeces* (*coprophagy*) – is performed by more individuals in our sample (prevalence: 0.85 vs. 0.49). Other studies present the fraction of activity budget spent in abnormal behaviour, calculated from durations or proportion of interval samples. By this measure, the individuals in our sample spent considerably more time behaving abnormally. Martin et al. [15] reported a median value of zero; four individuals who spent 5.6% of their time behaving abnormally were described as showing exceptionally high levels of abnormal behaviour. Similarly, Bloomsmith et al. [9] reported an average of only 0.3% (see also Hook et al. [4]). These figures are substantially lower than our average figure of 4.4%, and maximum of >60%.

Our study combined a relatively large sample of individuals across multiple populations with detailed, lengthy, direct, and continuous observations of behaviour. We found that some of the abnormal behaviours were performed for only short durations, or at low frequencies; many abnormal behaviours occurred rarely and only in limited contexts, and were often brief. Our choice of sampling method may have allowed us to record behaviours that might have been missed using other approaches. Continuous focal animal sampling allows the determination of prevalence, frequency, and duration, and so provides a more complete picture of the behaviour under investigation. For these reasons, this is the preferred sampling method for observational behavioural studies [39], [40] and we suggest future studies of abnormal behaviour should use this method when logistics allow.

So how abnormal is the behaviour of captive chimpanzees? In 1023 hours of focal animal sampling of wild chimpanzees in Uganda, none of the abnormal behaviours listed in this study were observed (Newton-Fisher, unpublished data). In contrast, seventeen of 37 behaviours we detailed in this study have been reported to occur (albeit rarely) in wild chimpanzees [20], [38]. The most prevalent form of ‘abnormal’ behaviour in this and other studies of captive populations – coprophagy – has been reported from at least six wild populations [42] and it may also be transmitted by social learning [29]. If this – or other – behaviour is ‘abnormal’ in captive chimpanzees, it may be the rate at which it is performed rather than simple occurrence that deviates from the behaviour of wild chimpanzees.

A formal comparison of rates and durations of these behaviour patterns between captive and wild populations is difficult: few sufficiently detailed quantitative data are available from wild populations, as might be expected for behaviours that are notably rare. The published literature on abnormal behaviour in wild chimpanzees is sparse [20], [42], [43] and rates of abnormality comparable to those that we describe here have never been reported. The hypothetical average individual in our sample of captive chimpanzees had a repertoire of five abnormal behaviours, performed some kind of abnormal behaviour once every forty minutes or so (although in real individuals, performance was more clumped than this figure implies) and spent between four and five percent of their activity in abnormal behaviours. Some individuals were far more extreme (over 60% of activity), and all individuals showed at least 2 different types of abnormal behaviour (cf. [29]).

Our data support a conclusion that, while most behaviour of captive chimpanzees is ‘normal’ in the sense that it is behaviour seen in their wild counterparts, abnormal behaviour is endemic in captivity. For some individuals it may dominate much of their activity, but for the rest it is a persistent element of their everyday behaviour despite living in social groups in enriched environments. Note that we do not address here whether captive chimpanzees use their normal behaviour in a manner atypical of wild chimpanzees, which may be a further dimension to ‘abnormal’ behaviour [44], [45].

In the wild, chimpanzee communities have a fluid social structure in which individuals are free to choose associates, mates, and ranging area (although all of these are subject to competitive effects). Similarly, they choose when, where, and what to eat; their natural diets include many species of flora and fauna, and they use a large variety of foraging, food processing, and hunting methods. Their daily activities vary accordingly, and they range widely over varied landscapes and habitat types [20], [46], [47], [48], [49]. In comparison, zoo-living chimpanzees have little opportunity to adjust association patterns, occupy restricted and barren spaces compared to the natural habitat, and have large parts of their lives substantially managed by humans [14]. Controlled diets and provisioned feeding contrast radically with the ever-changing foraging and decision-making processes of daily life in the wild.

Providing captive apes with more naturalistic enclosures, unpredictable feeding schedules and extractive foraging opportunities, as well as the opportunity to interact with conspecifics through housing in social groups, all appear to decrease the performance of abnormal behaviours [9], [13], [45], [50], [51]. Even the best zoo environments (which includes all zoos in this study) are limited, however, in what they can provide [13]. Some abnormal behaviours persist despite interventions to ‘naturalise’ the captive conditions and we suggest that captivity itself may be fundamental as a causal factor in the presence of persistent, low-level, abnormal behaviour (and potentially more extreme levels in some individuals). The cognitive and behavioural challenges in captivity are fewer than in the wild – stressful and dangerous place that it may be – and many normal behaviours and normal development are precluded. While extreme levels of abnormal behaviour may be explicable by individuals' particular histories, the pattern of low-level, pervasive abnormal behaviour shown by this study suggests that chimpanzee minds struggle to cope with conditions of captivity, despite the best efforts of those charged with their care.

From the perspective of human psychiatry, some of the behavioural abnormalities demonstrated by chimpanzees might be seen as symptoms of compromised mental health, i.e. mental illness, if they were observed in human primates [11], [17]. As with humans, chimpanzees are self-aware [52] sentient, emotional creatures [20], [53] with the potential to suffer [20], [54]. The apparently pervasive nature of abnormal behaviour, and its persistence in the face of environmental enrichment and social group housing, raises the concern that at least some examples of such behaviour are indicative of possible mental health problems [5], [11], [17].

Future research should address preventative or remedial actions, whether intervention is best aimed at the environment and/or the individual, and how to best monitor recovery [7]. More critically, however, we need to understand how the chimpanzee mind copes with captivity, an issue with both scientific [55] and welfare implications that will impact potential discussions concerning whether such species should be kept in captivity at all.
